# The effect of transcranial direct current stimulation combined with functional task training on motor recovery in stroke patients

**DOI:** 10.1097/MD.0000000000024718

**Published:** 2021-02-12

**Authors:** Fawaz Al-Hussain, Eman Nasim, Muhammad Iqbal, Nouf Altwaijri, Niaz Asim, Woo-Kyoung Yoo, Shahid Bashir

**Affiliations:** a Department of Neurology, Faculty of Medicine, King Saud University, Riyadh; b Neuroscience Center, King Fahad Specialist Hospital Dammam, Dammam, Saudi Arabia; c Department of Physiology, Faculty of Medicine, King Saud University, Riyadh; d Department of Physical Medicine and Rehabilitation, Hallym University Sacred Heart Hospital; e Hallym Institute for Translational Genomics & Bioinformatics, Hallym University College of Medicine.

**Keywords:** constraint-induced movement therapy, motor deficits, neurorehabilitation, stroke, transcranial direct current stimulation

## Abstract

**Background:**

Motor deficits are common after stroke and are a major contributor to stroke-related disability and the potential for long-lasting neurobiological consequences of stroke remains unresolved. There are only a few treatments available for the improvement of motor function in stroke patients. However, the mechanisms underlying stroke recovery remain poorly understood, and effective neurorehabilitation interventions remain insufficiently proven for widespread implementation.

**Methods:**

Herein, we propose to enhance the effects of brain plasticity using a powerful noninvasive technique for brain modulation consisting of navigated transcranial magnetic stimulation (TMS) priming with transcranial direct current stimulation (tDCS) in combination with motor-training-like constraint-induced movement therapy (CIMT). Our hypothesis is that navigated low-frequency rTMS stimulus priming with precise location provided by neuronavigation on the healthy side of the brain and with anodal tDCS on the affected side combined with CIMT will induce a greater motor function improvement than that obtained with sham tDCS combined with CIMT alone. We predict that the application of this technique will result in a large reduction in cortical excitability and dis-inhibition in the affected hemisphere and lead to improvements in behavioral measures of hand function in stroke patients.

**Discussion:**

The proposed study, therefore, is important for several reasons. The results could potentially lead to improved stroke therapeutics, and the approach makes use of 2 potential pathways to modulate brain function.

**Trial registration:**

This study protocol was registered in Clinical Trials Registry (https://clinicaltrials.gov/ct2/show/NCT04646577).

**Ethics and dissemination:**

The study has been reviewed and approved by the Human Research Ethics Committee of the King Fahad Specialist Hospital Dammam. The results will be actively disseminated through peer-reviewed journals, conference presentations, social media, broadcast media, print media, the internet and various community/stakeholder engagement activities.

## 1 Introduction

### 1.1 Rising incidence and burden of stroke

Stroke is a primary cause of death and disability globally. It is caused by an interrupted blood flow due to thrombosis or embolism to the brain which results in loss of vision, sudden paralysis, and impaired speech. In less than 15% cases, stroke leads to hemorrhage or cardiac arrest. According to the Global Burden of Diseases, Injuries, and Risk Factors Study (GBD), stroke was responsible for 5.5 million deaths after ischemic heart disease globally (1). In United States, stroke occurs once every 40 s and someone dies every 4 minutes, with death rate estimated to 41.6% in 2007.^[1]^ The absolute numbers of stroke-affected people may increase due to aging population. It is not only the high mortality; 50% survivors suffer from chronic disability. Thus, stroke has serious economic and public health significance. It is estimated that stroke cost 73.7 billion dollars in 2010 in United States and is projected to be 1.52 trillion dollars in 2050 (in 2005 dollars).^[1]^ Weakness, caused by damage of cortical networks for movement and their projections and disuse-related mechanisms, is considered a major cause of disability.^[2]^ Impaired function is experienced in the upper limb of two third of stroke survivors.^[3]^ High blood pressure, the major risk factor for stroke, is very common in Kingdom of Saudi Arabia (KSA). Incidence of stroke in Saudi population is 29.9 per 100,000.^[4]^ It is the third leading cause of death and in many surviving patients; it is the devastating endpoint of cerebrovascular disease.^[2]^ The impact of acquired brain injuries, for example stroke, upon individuals, families, and society continues to increase due to both the aging of the general population and the increasing length of post-insult survival. Stroke-related neurologic deficits, including motor function deficits, are often persistent, exerting a negative effect on the patient's quality of life.

### 1.2 Understanding stroke recovery mechanism based on brain plasticity is critical for developing new therapeutic approach

Brain plasticity is the ability of the brain to adapt to experiences, environmental pressure, and challenges which include brain damage.^[5,6]^ It happens within many levels, from molecules to cortical reorganization. There are multiple factors that influence the outcome of stroke, including the time after stroke, the lesion's location and the integrity of cortico-spinal tracts, and the cortical and subcortical connections. In general, each hemisphere undergoes neuroplasticity changes by means of regeneration, for example, axonal and dendritic sprouting with or without reorganization of cortical motor areas, for example, modulation of the synaptic plasticity or remapping of the functional representations by diminution of GABAergic inhibition, and increased NMDA receptor binding from lessoned areas onto the unaffected ipsilesional areas which surround the lesion or the homologous areas which are within the contralesional unaffected hemisphere.^[7,8]^

The latest literature specifies that noninvasive brain stimulation (NIBS) techniques (i.e. repetitive transcranial magnetic stimulation (rTMS) and transcranial direct current stimulation (tDCS)) can enhance the recovery of motor functions in chronic stroke patients. Two potential roles have been described for rTMS and transcranial direct current stimulation (tDCS) in stroke recovery:

(1) to inhibit, that is, downregulate, the healthy hemisphere, or
(2) to enhance excitability of the lesioned hemisphere.

Downregulation of the contralesional hemisphere can lead to improved motor performance in the affected limb, thought to be through a reduction in interhemispheric inhibition to the lesioned side. Most of the spontaneous stroke recovery after the acute phase entails plastic changes within the brain.^[9]^ For rehabilitation, new measures should be adopted to facilitate plasticity of brain which help in changes to occur more rapidly and more completely. Since plasticity in the hemisphere with stroke lesions leads to good recovery, one therapeutic approach such as brain stimulation should be focused to enhance brain plasticity in these areas.

### 1.3 Measurement of the cortical physiology and corticospinal tract integrity are important in terms of planning an effective treatment.

The progress in technologies which enable non-invasive exploration of the human brain have expanded our understanding of the reorganization of the brain after ischemic stroke.^[7–10]^ This kind of therapeutic approach solely relies on the functional and physiological status of each hemisphere and its balance between 2 hemispheres, which imply the importance of this physiological measure on planning an effective treatment strategy.

Cortical physiology could be measured by TMS using paired-pulse paradigm.

(1) short interval intracortical inhibition (SICI) is accepted to be GABAA-dependent since GABAA agonists increase SICI.
(2) long interval intracortical inhibition (LICI) is moderated by a long lasting GABAB-dependent IPSPs and the enabling of pre-synaptic GABAB receptors on inhibitory interneurons.
(3) Intracortical facilitation results from the net facilitation of mechanisms which are inhibitory and excitatory mediated by GABAA and NMDA receptors, respectively.
(4) Glutamatergic cortical interneurons could be involved in intra-cortical facilitation given that it is reduced by NMDA antagonists, for example, dextromethorphan. Those measurements will enable us to shed light on how the new therapeutic approach had effect in terms of inhibition and excitation balance in each and between hemisphere.

Diffusion tensor imaging tractography (DTI) is a new technique which enables non-invasive visualization of the human brain's fiber tracts in vivo^[10–13]^ which mostly has an impact on future design and choice of rehabilitation methods for each individual patient.

Measurement of the corticospinal tract volume, fractional anisotropy, mean diffusivity, axial diffusivity and radial diffusivity will give us insight about inter-individual variability of the efficacy of the therapeutic intervention.

Constraint-induced movement therapy (CIMT) is an intervention which has been used, for the most part, for treating upper extremities of stroke patients, which is based on perilesional reorganization.^[14,16]^ many of those successfully treated so far have been chronic patients that experienced stroke at least 1 year before starting CIMT therapy. It is approximated that patients whom are responsive to substantial improvement resulting from CIMT represent at least 50% of the stroke population in total^[17]^ current data advocate that CIMT therapy enhances motor function within a 2week period.

The effects of the treatment remain stable for many months after the end of therapy, and everyday lives of patients are adjusted accordingly with treatment effects.

Recently in neuroscience, NIBS techniques like (TMS or tDCS) have shown the potential to investigate causal brain-behavior relationships and for the rehabilitation of many diseases. It is important to mention that advances in neuroscience mostly depend on the union of evidence from multiple methods. Given that every technique has limitations of its own, combining various approaches will be, theoretically, a clear advantage.

### 1.4 Development of novel therapeutic approach combining motor learning and neuromodulation, which shared similar mechanism

As we know coupling cortical stimulation with motor learning might enhance motor recovery: As aforementioned, both strategies for enhancing motor recovery – motor learning and cortical stimulation - are associated with similar mechanisms of action – that is, increasing local excitability within the lesioned motor cortical area (possibly through synaptic strengthening) and decreasing it in the healthy contralateral hemisphere. Therefore we hypothesized that combining both therapies will enhance its individual effect on motor recovery. Indeed a few animal and human studies support such hypothesis. First, in an elegant study, Plautz et al noted that monkeys which were submitted to electrical stimulation which was subthreshold in combination with rehabilitative training multiple months after spontaneous recovery had improvements which were significant following therapy in motor performance, which persisted for several months.^[18]^ Also cortical mapping showed a large-scale surfacing of new hand representations within peri-infarct motor cortex, mainly within cortical tissue underlying the stimulating electrode.^[19]^ Second, a recent study showed that intensive rehabilitation therapy in combination with invasive epidural electrode has a significant improvement in motor function as compared with rehabilitation alone.^[20]^ Third, a recent study combining brain stimulation (rTMS) and motor learning (complex sequential finger motor task) showed that active motor cortex stimulation and motor training resulted in a significantly larger increase in motor cortex excitability in the affected hemisphere and enhanced motor performance accuracy as compared with motor training and sham stimulation.^[20]^ Therefore evidence supports that brain stimulation enhances the behavioral and neurophysiological effects of motor training in motor recovery.

There is sufficient evidence that NIBS induce neuroplastic changes which are mirrored in behavioral changes that can be observed. Albeit, the precise mechanism of NIBS in producing this neuromodulation in not well understood. Therefore, recent efforts to merge NIBS with neuroimaging in experimental paradigms were made to give a more methodical characterization of neuroplastic modulation by NIBS by way of using brain network analysis techniques.

### 1.5 Genetic influence on inter-individual variability, plasticity and behavior

Genetic polymorphism is also a contributing factor to affect the brain's response to disease and injury. Brain-derived neurotrophic factor (BDNF) is involved in the modulation of synaptic plasticity which is activity-dependent in the human motor cortex. A common single nucleotide polymorphism (BDNF val66met) in BDNF gene, has been associated with a decrease in the secretion of BDNF which then decreases the activity-related cortical plasticity following healthy individuals’ motor training and is associated with more errors and deficient retention in short-term motor learning.^[21,22]^

We therefore hypothesize that neuronavigation 1 Hz rTMS on health side, which provides real-time feedback and allows stimulation only when trajectory, coil position and coil rotation are within consistent, pre-determined boundaries, will be a more potent effect on the excitability of specific local cortical networks (identified during the optimal site assessment), prime with tDCS on affected side will enhance the effects of CIMT on motor function recovery in chronic stroke patients. Until recently the use of noninvasive brain stimulation coupled with motor learning was limited as rTMS or tDCS alone.

Currently, detailed information about stroke or brain plasticity is not available in the Middle East.

## 2 Methods

### 2.1 Overall strategy

Overall Approach, Participants, and Feasibility: We propose to study 40 patients (above 18 years) with first-time clinical ischemic or hemorrhagic cerebrovascular accident. Individuals with preexisting neurological or psychiatric conditions, those with unstable medical conditions, and those on any medication that might alter the TMS-tDCS measures will be excluded. A written consent will be obtained, and all the subjects will have full neurological and medical examination. Furthermore, detailed neuropsychological and behavioral assessment will also be performed in different domains. All participants will undergo a neurophysiologic study of local cortical reactivity, plasticity, and network dynamics using TMS-EMG following stroke. Participants will be randomized to active treatment with 1 Hz rTMS prime with real anodal or sham tDCS by means of randomization stratification. This approach guarantee that both groups have a close distribution in regards to the degree of paresis. Participants will be gathered into groups (strata) defined by the degree of paresis (low and high functional) and then randomized separately within each stratum according to a block randomization of 4 to receive real 1 Hz rTMS prime tDCS/CIMT and sham tDCS/CIMT. All participants will undergo brain magnetic resonance imaging (MRIs) for morphometric analysis and diffusion tensor imaging, and serial neurological and neuropsychological exams.

We have developed a safe and reliable method combining TMS with EMG to characterize cortical reactivity, plasticity, and brain network dynamics in humans.^[23–27]^

### 2.2 Brain MRI

Anatomical and DTI: In order to guide the TMS, all participants will undergo a brain MRI for brain lesions that are structural to generate a high-resolution anatomical brain image In addition, to examine structural brain integrity, T1 image volumes will be examined in 2 primary ways a) Reconstruction which is surface based and cortical thickness analysis b) An analysis that is volume based of the amygdala, hippocampal formation and different subcortical structures. Furthermore, a diffusion tensor imaging study (DTI) will be acquired using a diffusion-weighted (DW)-EPI sequence. Probtracx (FSL) will be used to delineate corticospinal tracts and corpus callosal connectivity.

### 2.3 Assessments – instruments of evaluation

A rater blinded to the treatment will assess the effects of the interventions using the instruments described below. Subject enrollment, consent checklist, and screening logs will be used as well as an inclusion and exclusion checklist to assist in prescreening measures. To assess treatment efficacy (motor function changes), we will use the following instruments:

(1) *The Jebsen Taylor Hand Function Test (JTHF)* - this test was designed as a broad measure of hand function. This test has been used by our and other groups to assess the effects of tDCS on motor function in stroke the (2) *Purdue Pegboard Test* which is a test that is more sensitive to measuring changes in finger dexterity. To characterize motor function at baseline, we will use the following instruments: (3) *Fugl Meyer Assessment of Motor Recovery:* this tool is a progressive assessment which measures balance, motor recovery, sensation and a few joint functions in individuals with hemiparesis. Also, it includes elements which address speed and coordination (4) *Modified Barthel Index Score (Activities of Daily Living):* this tool assess the disability degree in each individual. It records the independence indicators in regards to the disability caused by impairments. The score ranges from 0 to 20 and it is mainly concerned with physical aspects of disability. Finally, we will use (5) *the Modified Ashworth Scale:* this instrument is a 6-point rating scale that is used to measure muscle tone. In addition, we will use the *visual analog scale* for anxiety and the visual analog scale for Pain / Comfort. Finally to measure the adverse effects of the interventions of this study, we will use (6) the *Mini Mental State Examination (MMSE):* is a short screening tool go assess cognitive abilities. We will use it as a quick screening method to assess any cognitive alterations that could have occurred from before or after treatment or even at follow up. The consistent scores of MMSE can suggest that the patient had no changes in cognition throughout the intervention period, which could have affected the test performance or the continuation of the program. (7) Adverse event monitoring and reporting: At each session, the patient will be asked using open-ended questions about adverse events and (8) *Transcranial Magnetic/ Direct Current Stimulation Adverse Effects Questionnaire*: at each session, a questionnaire will be filled by the subject to assess potential common adverse effects of TMS/tDCS (redness at the site of stimulation, itching, neck pain and headache,) on a 5-point scale.

These instruments will be performed at the following time points by trained staff blinded to group assignment: during the week before the beginning of treatment (2) During day 5 and day 10 of treatment and (3) at 2 weeks of follow up, 4 weeks and 6 months after the end of treatment (Table 1). Moreover, to make sure that blinding is not broken within the duration of the study, we will use a questionnaire to assess the validation of the blinding at the end of the study. This questionnaire will ask both the participants and the rater whether the treatment was sham or active tDCS. The confidence of the responses will be rated accordingly, from 0 to 5, where 5 is total confidence and 0 is no confidence,

**Table 1:** Stroke and plasticity schedule of events.

	Screening visit	Training and tDCS Visits (10 d)			Follow-up visit
	1	2	6	10	4 wk
Consent	X				
Inclusion/Exclusion	X				
Pregnancy Test if indicated	X				
PMH/Physical	X				
Demographics	X				
Taylor Hand Function Test	X				X
Purdue Pegboard Test	X				X
Fugl-Meyer Assessment of Motor Recovery	X				X
Modified Barthel Index	X				X
Modified Ashworth Scale	X				X
Visual analog scale	X				X
Mini Mental State Examination		X	X	X	X
TMS and tDCS Screen	X	X	X	X	X
Training		X	X	X	X
MRI	X		X		
Pre/Post TMS Side Effect Questionnaire		X	X	X	X

MRI = magnetic resonance imaging, tDCS = transcranial direct current stimulation, TMS = transcranial magnetic stimulation.

### 2.4 TMS methodology

Navigated Brain Stimulation (NBS) system (ANT, NLtm) will be used to deliver TMS by a figure-of-eight (F8) coil coupled with a Magpro stimulator (MagVenture, Denmark). Navigated TMS has the ability to online monitor the targeted cortical region, coil position, and orientation within and across sessions by using structural MRI. It delivers trial-by-trial information of an induced electric field for each TMS pulse and the localization of the induced current maxima. Data obtained by this system are reproducible, which is an essential aspect of this type of study. The display of induced currents and calculation will assist to overcome the constraints of TMS by targeting relevant structures of brain alone. As differences between different individuals in structure–function relationships, may change the induced currents and precision of the neuronavigation system, particularly in individuals with brain pathology.^[28]^

**The repetitive TMS paradigm** will be administered at 1 Hz frequency and consist of 2000 pulses delivered over a 30 minute period at 90% of the patient's motor threshold. These parameters are known to cause brain activity inhibition. The optimal stimulation site (First dorsal interosseus and APB muscles), active and resting threshold will be established in the unaffected and affected hemispheres. Assessment of EMG signals will be done at a rate of 5000 Hz. Cortical silent period (cSP) and resting and active motor threshold (RMT, AMT) will be analyzed according to the IFCN guidelines.

**Transcranial direct current stimulation (tDCS)** individuals participating were seated in a comfortable chair. The use of the StarStim NE noninvasive wireless tDCS/EEG neurotransmitter (NE Neuroelectrics, Barcelona, Spain) was used to deliver the direct current sequentially and record the EEG data. A placement of small Ag/AgCl gelled electrodes was done over the lesioned hemisphere (anodal) and area F3 (return electrode), with a surface contact area of 3.14 cm2 specific to the StarStimNE device (Pi electrodes, Neuroelectrics). A control box device, which is wirelessly connected to a computer and communicated with the NIC (version 1.2, Neuroelectrics), will be connected to the electrodes. throughout anodal stimulation, delivery of direct current took place from a current-control circuit at an intensity of 1.5 mA and maintained for 20 minutes. As for the sham stimulation, placement of the electrodes was done in the same position and individuals participating were given a short ramp up (20 s total up/down) at the start and end of the stimulation period.

**Assessment of cortical excitability** – single and paired pulse TMS We will investigate changes in cortical excitability evaluating the motor evoked potential and the resting motor threshold (we will use the same methods as in our previous study, using the technique of paired-pulse for intracortical excitability and transcallosal inhibition in order to measure interhemispheric differences.^[13]^ Both the affected and unaffected primary motor cortex will be studied. We will use the following inter-stimulus interval – 2, 4, 6, 10 and 12 ms. The percentage of inhibition or facilitation for each ISI before and after treatment will be calculated. To measure changes in transcallosal inhibition, a suprathreshold stimulus is applied to the motor cortex of 1 hemisphere, and ten milliseconds later a threshold stimulus is applied to the contralateral motor cortex. The percentages will be calculated for each stimulus for before and after treatment.

### 2.5 Constraint induced movement therapy (CIMT)

Each subject will receive 14 days (2-week period: 10 weekdays) of CIMT (using the same methodology as described in a recent multi-site clinical trial.^[15]^ furthermore, during the 10 weekdays of the treatment period, the individuals participating will come into the laboratory to train the affected arm for up to 6 hours a day, using a variety of tasks according to a behavioral technique termed “shaping.”

**Statistical power and data analysis Sample size** will be determined with reference to other studies that used similar endpoints. We assume that a standardized difference of 0.8 (the difference between groups divided by the standard deviation) is conservative to detect a clinically meaningful difference between groups (active 1 Hz rTMS prime with tDCS/CIMT and sham tDCS/CIMT). We will assume a type I error of 5% (alpha), a type 2 error of 10% (beta), therefore, the power is going to be 90%. A t-test will be used (Using the sample size calculation for a normal distributed population); where 15 individuals per arm will be needed (therefore, a total of 30). We expect a 20% rate of loss to follow up or drop out. Moreover, we will assume that individuals who drop out will not improve from the last measured point; therefore, the sample size will increase by 20% to 36 participants (18 per arm). Furthermore, in case of unexpected factors such as higher placebo response, we increased the sample size further to 40 patients.

**PROTECTION OF HUMAN SUBJECTS** This study aims to enroll adult patients with a history of cerebral infarct that is chronic and associated with stable and significant motor deficits. Both ischemic and hemorrhagic stroke subtypes will be included. The entry criteria for patient enrollment are the following:

(1) age 18 to 90;
(2) first-time clinical ischemic or hemorrhagic cerebrovascular accident as noted in the radiological (or physician's) report;
(3) the ability to extend =20° at the wrist and 10° at the metacarpophalangeal and interphalangeal joints of all digits;
(4) participants must demonstrate adequate balance while wearing the restraint;
(5) the ability to stand from a sitting position and the ability to stand for at least 2 minutes with or without upper extremity support;
(6) weakness, defined as score of 15 to 55 (out of 66) on arm motor Fugl-Meyer scale; and
(7) stroke onset more then 6 months prior to study enrollment.

*Exclusion criteria*:

(1) having significant disability before stroke;
(2) any history of depression before the stroke (where appropriate);
(3) Any considerable decline in language reception, alertness or attention that might hinder with understanding the instructions for the motor testing;
(4) excessive pain in any joint of the paretic extremity;
(5) contraindications to single-pulse TMS (TMS will be used to measure cortical excitability) such as metal head implants;
(6) advanced cardiac, pulmonary, kidney or liver disease;
(7) a terminal medical diagnosis consistent with survival 1 year;
(8) coexistent major neurological or psychiatric disease (to decrease the number of confounders);
(9) a history of significant drug abuse in the prior 6 months;
(10) the use of certain neuropsychotropic drugs such as tricyclics, antidepressants, or 51 of 66 carbamazepine;
(11) active enrollment in a separate intervention study targeting stroke recovery;
(12) previously applied constraint-induced motor therapy and/or tDCS treatment for stroke; and
(13) a history of epilepsy before stroke (or episodes of seizures within the last six months).

### 2.6 Ethics

The study has been reviewed and approved by the Human Research Ethics Committee of the King Fahad Specialist Hospital Dammam (NEU329). The results will be distributed actively through journals which are peer-reviewed, conference presentations, broadcast, social and print media, the internet and multiple community/stakeholder engagement activities.

## 3 Discussion

Our proposed project is designed to challenge the current research and shift it with the clinical practice ideals; therefore, revise the current clinical practice. We believe that our hypothesis contributes an innovative account of the different findings noted in stroke rehabilitation and a practical approach to understand the fundamental features of motor decline. Also, we believe that identifying and understanding the regulation and consequences of inefficient plasticity may enable novel plasticity-based therapeutic interventions in the future. Moreover, our approach can help in inspecting the homeostatic plasticity at the level of brain networks that is induced by NIBS; it is a vital phenomenon for the of application of NIBS in managing diseases that involve pathologically altered cortical excitability, such as stroke. If we obtain our proposed objectives, this research will allow for a transformation of the process of brain plasticity through developing evidence-based technologies and interventions directed at

(1) establishing markers for altered cortical plasticity and
(2) the modulation of plasticity to improve motor performance.

## 4 Conclusion

In summary, such combined approaches to studying and quantifying the neurophysiological processes associated with neuroplasticity are critical to help identify, monitor, and potentiate neuroplasticity, which is crucial for functional recovery in patients suffering from brain lesions such as stroke.

## Acknowledgments

We thank Mr. Brent for technical assistance.

## Author contributions

All listed authors developed different substantial activities. FA, WKY and SB design the experiment and generate hypothesis. MI, AN and NE were involved in drafting the manuscript. Each author participated sufficiently in writing and reviewing the manuscript. All authors read and approved

**Conceptualization:** Fawaz Al-Hussain, Woo-Kyoung Yoo, Shahid Bashir.

**Data curation:** Fawaz Al-Hussain, Eman Nasim, Muhammad Iqbal, Niaz Asim, Shahid Bashir.

**Investigation:** Fawaz Al-Hussain, Eman Nasim, Muhammad Iqbal, Shahid Bashir.

**Methodology:** Fawaz Al-Hussain, Muhammad Iqbal, Nouf Altwaijri, Niaz Asim, Shahid Bashir.

**Writing – original draft:** Fawaz Al-Hussain, Eman Nasim, Muhammad Iqbal, Nouf Altwaijri, Niaz Asim, Woo-Kyoung Yoo, Shahid Bashir.

**Writing – review & editing:** Fawaz Al-Hussain, Eman Nasim, Muhammad Iqbal, Nouf Altwaijri, Niaz Asim, Woo-Kyoung Yoo, Shahid Bashir.

## Abbreviations

Abbreviations: MRI = magnetic resonance imaging, NIBS = non-invasive brain stimulation.

## References

[1] GBD 2016 Stroke Collaborators. Global, regional, and national burden of stroke, 1990-2016: a systematic analysis for the Global Burden of Disease Study 2016. Lancet Neurol 2019;18:439–58. 30871944 10.1016/S1474-4422(19)30034-1 PMC6494974

[2] McComas AJ Sica R E Upton A R. Functional changes in motoneurones of hemiparetic patients. J Neurol Neurosurg Psychiatry 1973;36:183–93. 4350702 10.1136/jnnp.36.2.183 PMC1083552

[3] Jørgensen HS Nakayama H Raaschou HO. The effect of a stroke unit: reductions in mortality, discharge rate to nursing home, length of hospital stay, and cost. A community based study. Stroke 1995;26:1178–82. 7604410 10.1161/01.str.26.7.1178

[4] Al Khathaami AM Algahtani H Alwabel A. The status of acute stroke care in Saudi Arabia: an urgent call for action!. Int J Stroke 2011;6:75–6. 21205244 10.1111/j.1747-4949.2010.00542.x

[5] Pascual-Leone A Amedi A Fregni F. The plastic human brain cortex. Annu Rev Neurosci 2005;28:377–401. 16022601 10.1146/annurev.neuro.27.070203.144216

[6] Nithianantharajah J Hannan AJ. Enriched environments, experience-dependent plasticity and disorders of the nervous system. Nat Rev Neurosci 2006;7:697–709. 16924259 10.1038/nrn1970

[7] Lima RR Santana LN Fernandes RM. Neurodegeneration and glial response after acute striatal stroke: histological basis for neuroprotective studies. Oxid Med Cell Longev 2016;2016:3173564. 28090244 10.1155/2016/3173564 PMC5165163

[8] Ward NS Newton JM Swayne OB. Motor system activation after subcortical stroke depends on corticospinal system integrity. Brain 2006;129:809–19. 16421171 10.1093/brain/awl002 PMC3717515

[9] Revathy Guruswamy Ayman ElAli. Complex roles of microglial cells in ischemic stroke pathobiology: new insights and future directions. Int J Mol Sci 2017;18:496. 28245599 10.3390/ijms18030496 PMC5372512

[10] Anke Karabanov Axel Thielscher Hartwig Roman Siebner. Transcranial brain stimulation: closing the loop between brain and stimulation. Curr Opin Neurol 2016;29:397–404. 27224087 10.1097/WCO.0000000000000342 PMC4947546

[11] Thickbroom GW Cortes M Avrielle Rykman. Stroke subtype and motor impairment influence contralesional excitability. Neurology 2015;85:517–20. 26187228 10.1212/WNL.0000000000001828 PMC4540249

[12] Dutta A. Bidirectional interactions between neuronal and hemodynamic responses to transcranial direct current stimulation (tDCS): challenges for brain-state dependent tDCS. Front Syst Neurosci 2015;9:107. 26321925 10.3389/fnsys.2015.00107 PMC4530593

[13] Bashir S Vernet M Najib U. Enhanced motor function and its neurophysiological correlates after navigated low-frequency repetitive transcranial magnetic stimulation over the contralesional motor cortex in stroke. Restor Neurol Neurosci 2016;34:677–89. 27567763 10.3233/RNN-140460 PMC5384333

[14] Newton JM Ward NS Parker CJM. Non-invasive mapping of corticofugal fibres from multiple motor areas – relevance to stroke recovery. Brain 2006;129:1844–58. 16702192 10.1093/brain/awl106 PMC3718077

[15] Nair DG Hutchinson S Fregni F. Imaging corre- lates of motor recovery from cerebral infarction and their physiological significance in well-recovered patients. Neuroimage 2007;3:253–63. 10.1016/j.neuroimage.2006.09.010 PMC2577311 17070707

[16] Richards LG Stewart KC Woodbury ML. Movement-dependent stroke recovery: a systematic review and meta-analysis of TMS and fMRI evidence. Neuropsychologia 2008;46:3–11. 17904594 10.1016/j.neuropsychologia.2007.08.013 PMC2248459

[17] Gong G He Y Concha L. Mapping anatomical connectivity patterns of human cerebral cortex using in vivo diffusion tensor imaging tractography. Cereb Cortex 2009;19:524–36. 18567609 10.1093/cercor/bhn102 PMC2722790

[18] Pannek K Chalk JB Finnigan S. Dynamic corticospinal white matter connectivity changes during stroke recovery: a diffusion tensor probabilistic tracto- graphy study. J Magn Reson Imaging 2009;29:529–36. 19243034 10.1002/jmri.21627

[19] Lindenberg R Renga V Zhu LL. Structural integrity of corticospinal motor fibers predicts motor impairment in chronic stroke. Neurology 2010;74:280–7. 20101033 10.1212/WNL.0b013e3181ccc6d9 PMC3122304

[20] Nitsche MA Cohen LG Wassermann EM. Transcranial direct current stimulation: state of the art 2008. Brain Stimul 2008;1:206–23. 20633386 10.1016/j.brs.2008.06.004

[21] Taub E Crago JE Uswatte G. Constraint-induced movement therapy: a new approach to treatment in physical rehabilitation. Rehabil Psychol 1998;43:152–70.

[22] Duncan PW. Synthesis of intervention trials to improve motor recovery following stroke. Top Stroke Rehabil 1997;3:1–20. 27620372 10.1080/10749357.1997.11754126

[23] Rossi S Hallett M Rossini PM. Pascual-Leone A; Safety of TMS Consensus Group. Safety, ethical considerations, and application guidelines for the use of transcranial magnetic stimulation in clinical practice and research. Clin Neurophysiol 2009;120:2008–39. 19833552 10.1016/j.clinph.2009.08.016 PMC3260536

[24] Bashir S Edwards D Pascual-Leone A. Neuro-navigation increases the physiologic and behavioral effects of low-frequency rTMS of primary motor cortex in healthy subjects. Brain topography 2010;24:54–64. 21076861 10.1007/s10548-010-0165-7 PMC3589808

[25] Bashir S Perez J Horvath JC. Differentiation of motor cortical representation of hand muscles by navigated mapping of optimal TMS current directions in healthy subjects. J Clin Neurophysiol 2013;30:390. 23912579 10.1097/WNP.0b013e31829dda6b PMC3740163

[26] Bashir S Yoo WK Kim HS. The Number of Pulses Needed to Measure Corticospinal Excitability by Navigated Transcranial Magnetic Stimulation: Eyes Open vs. Close Condition Front Hum Neurosci 2017;11:121 doi: 10.3389/fnhum.2017.00121. 28377705 10.3389/fnhum.2017.00121 PMC5359302

[27] Bashir S Vernet M Yoo WK. Changes in cortical plasticity after mild traumatic brain injury. Restor Neurol Neurosci 2012;30:277–82. 22596356 10.3233/RNN-2012-110207 PMC3951777

[28] Al-Sultan F Al-Zahrani A Al-Kahtani F. The future of transcranial magnetic stimulation in neuroscience and neurology in the Middle East. Eur Rev Med Pharmacol Sci 2019;23:4354–9. 31173309 10.26355/eurrev_201905_17942

